# High-speed resistance training and balance training for people with knee osteoarthritis to reduce falls risk: study protocol for a pilot randomized controlled trial

**DOI:** 10.1186/s13063-017-2129-7

**Published:** 2017-08-18

**Authors:** Pazit Levinger, Jeremy Dunn, Nancy Bifera, Michael Butson, George Elias, Keith D. Hill

**Affiliations:** 1 Institute of Sport, Exercise and Active Living (ISEAL), College of Sport and Exercise Science, Victoria University, PO Box 14428, Melbourne, VIC 8001 Australia; 2 School of Physiotherapy and Exercise Science, Curtin University, Perth, WA Australia

**Keywords:** Osteoarthritis, Falls, Balance, Power, Resistance training

## Abstract

**Background:**

The number of falls experienced by people with knee osteoarthritis (OA) is almost double the number experienced by people with no OA. The neuromuscular elements required to arrest a fall are more impaired in people with knee OA compared to their asymptomatic counterparts. Therefore, these elements may need to be incorporated into an exercise intervention to reduce the risk of falling. The aim of this study will be to examine the feasibility, safety and patient satisfaction of a high-speed resistance-training program, with and without balance exercises, in people with knee OA compared to a control group. The effect of these exercise programs on lower-limb muscle strength and physiological and functional risk factors for falls will also be examined.

**Methods:**

This study will be a pilot randomized controlled trial with a pre- and post-intervention design (outcome assessments at baseline and 8 weeks after participation commencement) comparing three groups: a control group (no intervention), a high-speed resistance-training group and a high-speed resistance-training plus balance exercises group. Thirty people with knee osteoarthritis aged 60–90 years will be recruited and randomized to one of the three groups. Feasibility and safety will be assessed by examining adherence to the exercise program, dropout rate, pain level during and following exercise, number of exercises stopped due to pain, and any adverse event or any incident that prevents the participant from completing the prescribed exercise. Secondary measures of lower-limb strength, physical function, self-reported pain and function, fear of falls, and executive function and quality of life will also be assessed. To determine statistical trends of effectiveness and hence to inform sample size for a fully powered study, analyses of the secondary outcomes will be performed to assess the changes within and between groups over time (pre-post) using repeated measure ANOVA.

**Discussion:**

The results of this study will improve understanding of what type of exercise is safe and beneficial for people with knee OA to reduce their risk of falling, and hence will inform the development of a future large research trial.

**Trial registration:**

Australian New Zealand Clinical Trials Registry, ID: ACTRN12616001382460. Registered on 6 October 2016.

**Electronic supplementary material:**

The online version of this article (doi:10.1186/s13063-017-2129-7) contains supplementary material, which is available to authorized users.

## Background

Falls in older people are a major public health problem with substantial economic health cost and significant implications for the individuals. Knee osteoarthritis (OA), a common painful, debilitating and life-altering joint disease, is present in 50% of adults over 65 years of age. The prevalence of falls in people with OA is substantially higher than in older individuals generally (30%), with approximately 50–60% of those with knee OA over the age of 60 years reporting one or more falls each year [1–3]. However, despite the high prevalence of falls in people with knee OA, the mechanism of falling in this group is unclear. Moreover, there are no randomized controlled trials demonstrating the effectiveness of different types of exercise or other fall-prevention approaches in reducing falls in people with OA.

Aging is associated with neuromuscular changes that affect the ability to maintain dynamic postural control during imbalance episodes such as slower muscle response, poorer muscle strength and muscle power. In people with knee OA, these neuromuscular deficits might be more impaired, leading to a greater risk of falling when rapid balance recovery responses are required such as when falling. In our previous research, we found that when simulating a forward fall, those with knee OA demonstrated slower step responses and an impaired ability of the knee joint to absorb impact forces and slow down the body’s forward momentum to arrest a fall [4]. These biomechanical responses observed in people with knee OA might be related to loss of muscle power, especially the quadriceps, which is the most commonly affected muscle in this patient group [5]. Muscle power reflects the ability of the muscle to produce force rapidly. Given that recovering balance requires a rapid response, exercises that target fast muscle activation might be effective in maintaining adequate muscle function to prevent falls in older people and in those with knee OA.

Exercise interventions that focus on balance exercises have been shown to be effective in reducing falls for older people [6]. However, there is no evidence to suggest that current fall-prevention programs will be effective for people with knee OA. Moreover, existing exercise-based fall-prevention programs mainly focus on maintenance of balance and do not focus on training the neuromuscular elements required to arrest a fall. The neuromuscular elements required to arrest a fall are more impaired in people with knee OA compared to their asymptomatic counterparts [4]. To recover balance from falling, adequate muscle force and joint power generation is required [7]. This is adversely affected by OA. Hence, these unique factors and the associated pain must be taken into account in the design of fall-prevention strategies in this high-risk group. Consequently, development of an exercise approach aimed at reducing falls that is specifically designed to address these neuromuscular factors for people with knee OA is essential.

In this study we aim to examine the feasibility, safety and patient satisfaction of a high-speed resistance-training program, with and without balance exercises, in people with knee OA compared to a control group. Given the pain associated with knee OA, it is crucial to determine what effect high-speed resistance training that focuses on muscle power has on knee pain. This is of particular importance as this mode of exercise may potentially increase impact load on the knee joint due to the rapid and powerful movements involved. Therefore, feasibility and safety of the proposed exercise intervention must be assessed to determine if this exercise approach is safe to prescribe for those with knee OA. The effect of the exercise interventions on strength, physiological and functional risk factors for falls, pain, and executive function will also be assessed to provide data to support a sample size calculation for a fully powered trial.

## Methods

All procedures involved in this trial will be conducted in compliance with the National Statement on Ethical Human Resource and the Australian Code for the Responsible Conduct of Research. Ethical approval has been obtained from the Human Research Ethics Committee from Victoria University, Melbourne (Application ID: HRE15-285). All participants will provide written informed consent. The study was designed according to the Consolidated Standard of Reporting Trials (CONSORT) guidelines and the Standard Protocol Items: Recommendations for Interventional Trials (SPIRIT) 2013 Checklist (see Additional file 1) and publications associated with the trial will be reported according the CONSORT 2010 Statement [8]. The trial has been retrospectively registered in the Australian New Zealand Clinical Trials Registry (ACTRN12616001382460).

### Design and setting

This study is a pilot randomized controlled trial with a pre- and post-intervention design (outcome assessments at baseline and at 8 weeks after participation commencement) comparing three groups: a control group (no intervention), a high-speed resistance-training program and a high-speed resistance-training plus balance exercises program. The groups will be assessed before and after the 8-week program to examine the effect of the intervention on pain, adherence to the intervention, lower-limb muscle strength, physiological and functional risk factors for falls, executive function, fear of falling, quality of life and participant perceptions of the interventions.

#### Participants

Participants aged 60–90 years with knee OA who have had a fall in the previous 12 months, or who are concerned about having a fall, will be recruited. Clinical diagnosis of knee OA based on the American College of Rheumatology will be used [9].

Inclusion criteria: participants will need to have had knee pain for at least 6 months and experience current average pain of at least 3 (on an 11-point Numerical Pain Rating Scale (NRS)) and be able to ambulate independently (with no more than a single-point stick used for ambulation). A total of 30 participants will be recruited (10 per group).

In addition to the above criteria, participants will also have to have one of the following criteria indicating increased risk of falling: (1) have had at least one fall in the past 12 months and (2) have limited their activity level due to concern about falling. Including people who are concerned about falling (with or without falls) will increase recruitment, and maintain a group at increased risk of falls, as activity limitation is often associated with reduced balance and mobility [10].

Participants will be excluded from this study if they have: (1) any uncontrolled nonmusculoskeletal conditions that would make testing difficult and uncomfortable such as chronic obstructive airways disease and/or congestive heart failure, (2) a pre-existing neurological condition that affects lower-limb strength, balance and or ambulation (e.g., polio, stroke), (3) any uncontrolled musculoskeletal or orthopedic conditions that may affect ambulation (e.g rheumatoid arthritis), (4) current involvement in a structured resistance-training and/or an organized balance-training program more than once a week, (5) any documented medical condition or physical impairment that is deemed by the medical practitioner to contraindicate their inclusion and (6) mild cognitive impairment or dementia determined by a score of less than 25 using the Saint Louis University Mental Status (SLUMS) test [11].

### Procedure

**Fig. 1:**
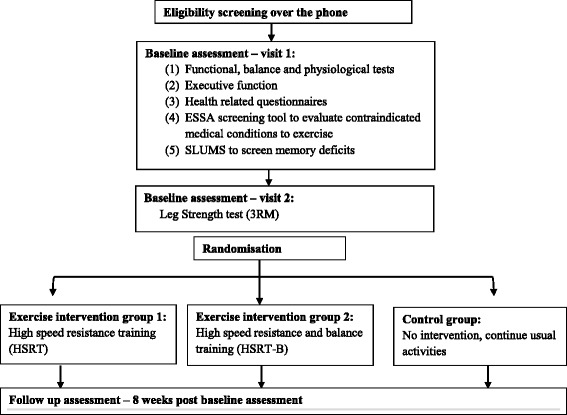
Flow diagram of study procedure, recruitments and randomisation

**Fig. 2:**
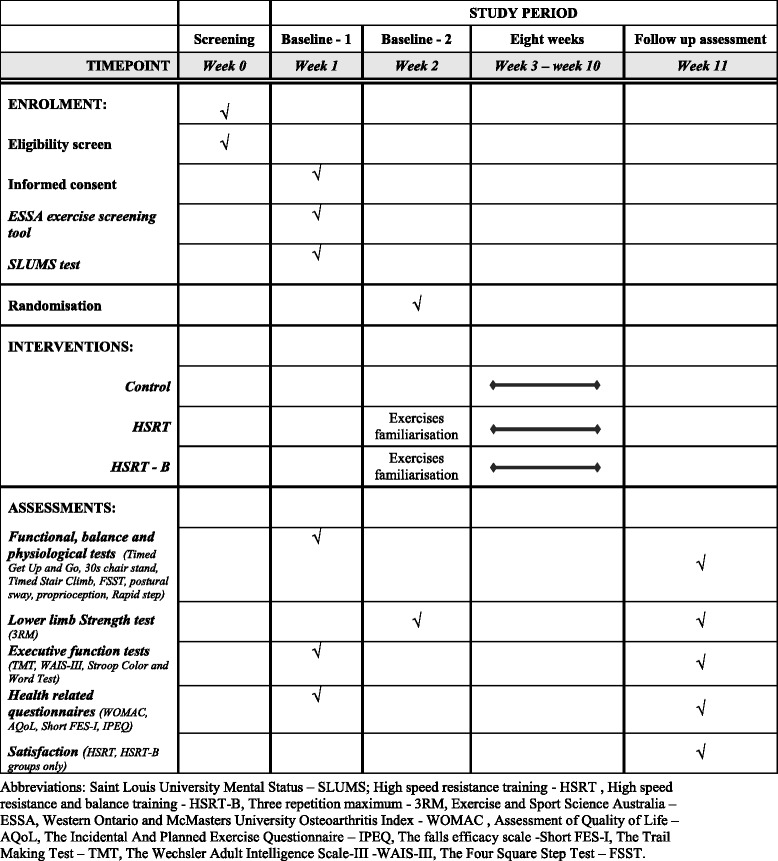
Study design schedule in accordance with the Standard Protocol Items: Recommendations for Interventional Trials (SPIRIT) Figure

The procedure is outlined in Fig. 1. Preliminary screening will be conducted over the telephone by one of the researchers. Volunteers who are deemed eligible will be scheduled a first visit. All participants will undergo the following physiological and functional assessments including lower-limb strength and proprioception, functional and balance tasks and executive function tests. Moreover, measures of physical activity, quality of life, pain and function, fear of falls, as well as falls history, will also be recorded. Participants’ satisfaction with the exercise program and their perception of improvement will also be assessed at the follow-up visit at the completion of the 8-week intervention. Figure 2 outlines the study design schedule in accordance with the SPIRIT Figure.

Participants will first attend the Victoria University Footscray Park campus for baseline testing. During the first visit all measures, excluding strength, will be assessed. Familiarisation with the strength test will be conducted in the first visit followed by actual testing during a second visit (approximately 3–6 days apart). A set of questions to assess cognitive impairment (memory) will also be conducted in the initial visit using the SLUMS test where participants who score below 25 cut-off scores will be excluded from the study [11]. In the second visit, after completion of the strength test, participants will be randomized to one of the three groups: (1) high-speed resistance-training (HSRT) or (2) high-speed resistance-training and balance exercises (HSRTB) or (3) control group (no intervention). All eligible participants will complete the Exercise and Sport Science Australia (ESSA) exercise screening tool to evaluate any contraindicated medical conditions to exercise (https://www.essa.org.au/wp-content/uploads/2011/09/Screen-tool-version-v1.1.pdf). Participants answering “yes” to any of the screening questions will be required to obtain medical clearance from their local physician prior to participation in the intervention. Only participants randomized to one of the exercise intervention groups will need to obtain medical clearance if deemed relevant. Participants in the exercise intervention groups will attend Victoria University Exercise Rehabilitation Clinic twice a week for 8 weeks (total of 16 exercise sessions). Participants from the control group will be advised to continue with their usual activities. All participants will be reassessed after 8 weeks from the baseline assessment.

## Recruitment

Participants will be recruited from the general community and from the western suburbs of Melbourne. Advertisements in local newspapers will be used for recruitment. In addition some participants will be recruited from the staff body at Victoria University through advertisements in University publications and posters displayed on notice boards, as well as global emails to staff and students, and social media. Advertisements will also be placed in health care facilities and places with high circulation of senior citizens and will also be mailed-out to health care practitioners in Melbourne.

### Randomisation

All participants will be randomly allocated to one of the following groups: (1) HSRT or (2) HSRTB or (3) control group (no intervention). Block randomisation will be undertaken using opaque envelopes, so that blocks of six to seven participants (two to three for each intervention group and two to three for control group) will be randomized at a time. As participants will either be doing one of two different exercise programs or not doing an exercise program, assessors and participants will not be blinded to their respective group allocation.

### Exercise interventions

#### High-speed resistance training (HSRT)

Participants who are randomized to the HRST will receive an exercise program that includes six to eight exercises targeting the lower limbs, to be performed twice a week for 8 weeks. The exercise sessions will be delivered by a qualified exercise physiologist or physiotherapist. The exercise program will include the following exercises: leg press, squat, step-up, lunges, going up stairs, sit to stand, calf raises (Table 1). Participants will be instructed to perform the exercises in a rapid and explosive manner such that all repetitions for each shortening phase (concentric activation of the muscle) will be performed as quickly as possible while the lengthening phase of the muscle (eccentric phase) will be controlled over 2–3 s. Several previous research trials have used similar principles of a high-speed training program for older people and those with knee OA and reported the training to be beneficial for older people, safe and with no adverse events [12–14]. However, none of the above studies investigated the effect of high-speed power training on balance or physiological risk factors for falls in this patient group, nor did they include functional weight-bearing exercises.

**Table 1:** Details of the exercises and progression of the high-speed resistance program in the three phases

Exercise	% 1RM	Sets × repetitions	Progression
Phase 1 – week 1–2 (4 sessions)			
Bilateral leg press	20–40%	2 × 8–12	Load to be increased by 5–10% (of 1RM or % of BW) once participant reaches the upper limit of repetitions during their last set of each exercise with correct technique and velocity of contraction. All weight-bearing exercises will begin using bodyweight only ^a^height of chair/step adjusted to participant’s ability
^a^Step up		2 × 8–12	
^a^Sit to stand		2 × 8–12	
Calf raise		2 × 8–12	
Phase 2 – week 3–5 (6 sessions)			
Bilateral leg press	40–60%	2 × 5–8	Load to be increased by 5–10% (of 1RM or % of BW) once participant reaches the upper limit of repetitions during their last set of each exercise with correct technique and velocity of contraction. All weight-bearing exercises will begin using bodyweight only
^a^Step up		2 × 5–8	
^a^Sit to stand		2 × 5–8	
Calf raise			
^b^Squat		2 × 5–8	
^b^Stair climbing		2 × 5–8	
^b^Lunge		2 × 5–8	
Phase 3 – week 6–8 (6 sessions)			
Bilateral leg press	60–80	2–3 × 2–5	Load to be increased by 5–10% (of 1RM or % of BW) once participant reaches the upper limit of repetitions during their last set of each exercise with correct technique and velocity of contraction. All weight-bearing exercises will begin using bodyweight only
Step up		2–3 × 2–5	
Sit to stand		2–3 × 2–5	
Calf raise		2–3 × 2–5	
^b^Squat		2–3 × 2–5	
^b^Stair climbing		2–3 × 2–5	
^b^Lunge		2–3 × 2–5	

*1RM*, one repetition maximum, *BW* body weight

^a^height of chair/step adjusted to participant’s ability

^b^exercises to be added if participant is able to perform safely (in Phase 2 from week 4 onward)

#### High-speed resistance and balance training (HSRTB)

Participants who are randomized to the HRSTB will receive the same exercises as the HSRT group with the addition of six balance exercises. The balance exercises will include the following: walking forward and backward, single leg standing, single leg tapping, side stepping and backward walking. Progression of the balance exercises will include decrease in base of support, decrease in hand support and reduced sensory input (e.g., eyes closed). Each balance exercise will initially be of 15–30 s duration and be performed twice per session. Balance exercises are detailed in Table 2.

**Table 2:** Details of the balance exercises and exercise progression

Exercise	Description	Sets/Reps/Time	Progression
1. Single leg balance – firm surface 10 s	Participant stands on one leg to balance with hands on hips Leg in air must not contact stance leg, floor or any other object to aid in balance	2 × 10 s	1. Increase time by 5 s increments (after performed same time 2 consecutive times) 2. Reach up to 30 s 3. Foam surface starting 10 s 4. Increase 5 s with progression 5. Repeat steps 1–4 commencing with eyes closed
2. Walking forward – tandem on firm surface (in between parallel bars)	Participant stands in between parallel bars Place one foot directly in front of the other (heel to toe) so they form a straight line	4 × lengths of parallel bars	1. Walking forward tandem on firm surface 2. Toe walking on firm surface turn on toes at end 3. Repeat steps 1–3 on foam 4. Repeat steps 1–3 eyes closed
3. Walking backwards – tandem on firm surface (in between parallel bars)	Participant stands in between parallel bars Place one foot directly behind the other (toe to heel) so they form a straight line	4 × lengths of parallel bars	1. Walking backward tandem on firm surface 2. Toe walking on firm surface 3. Repeat steps 1–3 on foam 4. Repeat steps 1–3 eyes closed
4. Side step walking (in between parallel bars)	Stand up tall and place hands on hips Take 10 side steps to the right Take 10 side steps to the left	4 × lengths of parallel bars	1. Side step walking firm surface 2. Side step walking with 1 obstacle in the middle 3. Side step walking with 2 obstacles 4. Continue to progress like this until every step is over an obstacle 5. Side steps with grapevine 6. Repeat steps 1–5 on foam 7. Repeat steps 1–5 with eyes closed
5. Single leg tap – clockwork (forward, lateral, behind) on firm surface	Standing on one leg Reach out to do clockwork starting with forward, lateral and backward tap Bring foot back down to standing Repeat starting from backward, lateral and forward tap.	2 × 5 forward, lateral, backward tap 2× backward, lateral, forward tap	1. 2 × 5 taps bringing foot back to midline in between each tap out 2. 2 × 5 taps not bringing foot back to midline 3. 2 × 5 and include tap on opposite side (cross shape) 4. Repeat steps 1–3 with stance foot on foam 5. Repeat steps 1–3 with eyes closed

Single leg exercises to be performed on each leg

## Exercise protocol and familiarisation sessions

Participants will undergo up to two familiarisation sessions prior to commencement of the intervention to ensure that they feel comfortable with the exercises and perform them with the correct technique. To determine the training load (resistance weight to be lifted) for the high-speed resistance-training exercises for each participant, an estimation of their one repetition maximum (or 1RM) will be calculated indirectly using submaximal estimation [15]. Participants will perform between 7 and 10 repetitions of each exercise with the maximum weight that they could lift. Participants performing the weight-bearing exercises (such as sit to stand, squat, calf raises) will initially use their body weight only. Additional weight (using a vest weight) will then be added as needed. Their estimated 1RM will be then used to determine the relative load/resistance required for the training protocol as detailed below.

### Exercise progression protocol

Each session will include a 5-min warm-up on a fitness bike or treadmill before commencement of the program and a 5-min cool-down of stretches of relevant muscles.

The 8-week program will be divided into the following phases to allow gradual progression and increase of the resistance for the high-speed resistance-training exercises (weight/training load) as detailed in Table 1:

- Phase 1 – weeks 1–2 (four sessions). Participants will perform two sets of 8–12 repetitions with 20–40% 1RM
- Phase 2 – weeks 3–5 (six sessions). Participants will perform two sets of 5–8 repetitions with 40–60% 1RM
- Phase 3 – weeks 6–8 (six sessions). Participants will perform two to three sets of 2-5 repetitions with 60–80% 1RM

The principle of progressing the exercise program will be similar to previous research in older people [11] as follows: for phases 1 and 2, the resistance/load will be increased by 5–10% of 1RM when the number of repetitions a participant will be able to perform (with correct technique at the required speed) will be more than eight in their last set. Similarly, in phase 3, the resistance/load will be increased (by 5–10% of 1RM) when the number of repetitions performed (with correct technique at the required speed) is five in the last set. Rest periods of 2–3 min will be given between sets.

Progression of the exercise will also be dependent on the individual perception of the difficulty of exercise (exercise intensity) and the level of pain as detailed below:

The 20-point Borg Rating of Perceived Exertion (RPE 6–20) scale will be used to determine the individual perception of difficulty of the exercises [16]. The RPE will be used to determine the intensity of each exercise where participants will be encouraged to exercise with a RPE of between 11 and 15/20. Increase in the training load will be made when a RPE of below 10/20 (“too easy/light”) will be reported by the participant.

The level of pain will also be recorded using the 0 to 10-point Numerical Pain Rating Scale (NRS; 0 –“no pain” to 10 – “worst possible pain”) for each exercise and will allow the load to be adjusted if the pain reported is too high [17]. Mild to moderate pain is expected in people with knee OA while exercising. However, substantial increases in pain may suggest that modifications to the exercise program are required [18]. A pain level of 7 and above will be deemed as too painful and load adjustment will be made.

### Pain-level documentation, exercise-stopping rules and adverse events

Pain level will be recorded using the NRS (0–10) during the sessions, 2 h and 24 h later. Any substantial increase in pain will be recorded. Pain-relief medication usage will be recorded via logbook by the participants during the intervention period.

The present study protocol will use similar principles to record pain and safety as reported in a previous dose-response trial in people with knee OA [19]. Substantial increases in pain will be defined as knee pain of at least 7/10 and or pain increased by at least 2–3/10 points (3/10 if initial pain is between 0 and 6/10 and 2/10 if initial pain is between 7 and 8/10) during the sessions, 2 h and 24 h after an exercise session as detailed below. The thresholds of 3/10 and 2/10 are based on a previous study suggesting that 2/10 represents a clinically significant change in pain and varies depending on the initial level [20].

An exercise-stopping rule will be defined as an incident (due to substantial increase in pain or an adverse event) that stopped the participant from completing the prescribed exercise for safety reasons and is likely to be related to the exercise program [19].

#### Substantial knee pain levels during an exercise session

A pain level increase during an exercise to more than 7 or an increase in 3 points (if initial pain is between 0 and 6/10) will result in reducing the exercise load. If, after reducing load, the pain level remains at 7 or above, the specific exercise will be stopped. The specific exercise will be attempted again in the next session and the same rules will be applied. If stopped again, an *adverse event* will be deemed to have occurred and the exercise will no longer be practiced by the participant.

#### Substantial knee pain levels after an exercise session (2 h and 24 h post session)

If a participant reports to the investigator a substantial amount of pain (as defined above) more than 2 h after an exercise session which lasts longer than 24 h, the exercise program will be reviewed and load will be reduced. If a substantial increase in pain occurs again in the next session, an *adverse event* will be deemed to have occurred and the exercise program will be reviewed and modified.

##### Serious adverse events

The following circumstances will be considered as serious adverse events: any report of difficulty in breathing that does not settle quickly with rest; new or unrelenting chest pain; or acute changes in level of consciousness during the session. A serious adverse event will be defined if symptoms have not settled and a medical emergency procedure will be required.

### Control group

During the 8 weeks, participants from the control group will be advised to continue with their usual activities. Usual activities will be defined as any normal day-to-day activities and or any current usage of health services.

## Outcome measures

### Feasibility and safety outcomes (primary measures)

Data for feasibility and safety will be analyzed as follows: percentage of participants who complete the intervention, overall percentage of sessions attended, number of participants who dropout due to reasons related to the exercise program or the overall research project, number of sessions and or number of exercises stopped due to pain, any adverse event or incident that stops the participant from completing the prescribed exercise.

## Secondary outcomes

Demographic information including falls history (and associated information surrounding circumstances of falls), duration of joint symptoms, comorbidities, medical conditions and medications will be collected.

The following functional, physiological and cognitive tests will be assessed at baseline and at the follow-up assessment. These selected tests are key measures used to identify risk of falls:

1. Leg strength and endurance will be assessed using the 30 s chair stand test. Participants will sit on a chair (a 43-cm high chair) and stand up, then repeat, as many times as they can for 30 s (no upper limb support), the number of sit to stand efforts performed will be recorded [21]
2. Physical function will be assessed using the Timed Get Up and Go Test (with and without dual tasks (manual and cognitive)) [22]. Participants will stand up from a chair, walk 3 m as quickly and safely as possible, cross a line marked on the floor, turn around and walk back and sit down. This test will also be performed when carrying a glass of water (manual dual task) and when counting backward by 3’s (cognitive dual task). The time taken to complete the task will be measured with a stopwatch. One practice trial will be given followed by an actual test
3. A rapid dynamic weight shift, coordination and stepping will be assessed using The Four Square Step Test (FSST) of balance [23]. Participants will step as fast as possible into four squares marked on the floor with tape (labeled 1–4) in a certain sequence (starting from square 1 then 2, 3, 4, 1, 4, 3, 2 and 1). This sequence requires the participant to step forward, backward, and sideways to the right and left. The score will be recorded as the time taken to complete the sequence
4. Functional muscle power will be assessed using the Timed Stair Climb Test (10 steps, 14-cm rise). The time taken to complete the task (climb 10 steps as quickly as possible) will be recorded and the stair climbing power will be calculated using a formula described in previous research [24]
5. Static balance will be assessed by evaluating postural sway of the body in the medio-lateral (M/L) and anterior posterior (A/P) directions using a swaymeter while standing on a medium-density foam rubber mat (70 × 60 × 15 cm thickness) with the eyes open and closed. The total excursion of the sway in each direction is measured in millimeters [25]
6. Knee proprioception will be measured during sitting by matching the position of the legs on either side of a clear plastic sheet with the eyes closed [3]. Any error in matching the limbs will be recorded in degrees. After two practice trials, the average of five trials will be recorded
7. The ability of older people to step maximally and rapidly in multiple directions will be assessed using the Maximum Step Length and Rapid Step Test [26]. Initially, the maximum step length in the front, side and back directions will be assessed. With arms crossed over the chest, the participant will step maximally with one leg while keeping the other leg planted and then to return to the initial position in one step. The test will be repeated for each leg in three directions (front, side, back). Three practice trials will be given followed by a series of five trials for each leg in each direction. The step length will be measured and the average of the five trials will be used for the rapid step test. For the rapid step test, marks on the floor will be placed at 80% of the maximum step length measured previously in each direction. Participants will then step as fast as possible with one leg in a given direction on the marks on the floor as instructed by the assessor. A practice set of four steps for each leg will be given. The actual testing will include a series of 24 steps randomly selected by the assessor which will include four steps in each direction for each leg. The total time taken to complete the step sequence and any errors will be measured. An error will be defined as (1) loss of balance, (2) failure to return to the initial position, (3) multiple steps and (4) noncompliance with direction or side
8. Lower-limb muscle strength will be assessed by having participants perform three maximal repetitions (3RM) during bilateral leg press using a seated leg-press machine. The maximum amount of weight lifted by participants through three full-range repetitions while maintaining correct technique will be used for determining the participant’s maximum 3RM strength. Participants will have a familiarisation session before the actual recording of the 3RM test. During the familiarisation session participants will be shown the correct exercise technique and be given the opportunity to familiarise themselves with the exercise. Initially, each participant will perform a warm-up set of eight repetitions on the leg press with a light load. This will then be followed by the completion of five to six repetitions at a heavier weight and rest period of 2–3 min. The load will be increased incrementally until only three repetitions with correct technique are able to be completed

### Cognitive tests – assessment of executive function

Exercise is associated with an improvement in cognitive performance [27], most notably executive functions. Executive function is a psychological construct used to describe cognitive processes that enable the formation, planning, execution and monitoring of goals. The processes involved include inhibitory control, working memory and cognitive flexibility [28]. The neuropsychological assessment instruments that will be used to assess executive functions of set switching, inhibition and updating/working memory [29, 30] will include *The Trail Making Test* (*TMT*) [31], *The Wechsler Adult Intelligence Scale-III* (*WAIS-III*) [32] and *The Stroop Color* and *Word Test Victoria Version* [33]. These tests are commonly used in research and have demonstrated reliability and validity for this age group.

1. Set-shifting and psychomotor speed will be assessed using The Trail Making Test (TMT). The TMT is a two-part (TMT-A and TMT-B) assessment of general brain function. The TMT-A requires participants to connect numbers with a line, while the TMT-B requires both letters and numbers to be connected. TMT-A assesses visuo-perceptual and motor abilities, while the TMT-B assesses working memory and task-switching ability [31]. Participants will be administered a pen-and-paper version of the TMT, which takes 5–10 min to complete
2. Working memory will be assessed using The Digit Span Task from The Wechsler Adult Intelligence Scale-III (WAIS-III). The WAIS-III is a widely used battery of tests that assesses different aspects of adult human intelligence [32]. Each task has a forward and backward version. In the Forward Digit Span Task the respondent is verbally presented with a string of numbers by the examiner and is asked to repeat them back exactly as they were presented. The string of numbers starts at two and continues until the respondent fails to repeat the correct sequence of numbers on both attempts. The Backwards Digit Span Task requires the respondent to repeat the string of numbers in the reverse order. Two trials of each span length will be attempted. The task is discontinued if the respondent fails to repeat the correct sequence on both attempts
3. Selective attention, response inhibition and speed of processing will be assessed using The Stroop Color and Word Test Victoria Version [33]. The test has three pages. The first page has colour names printed in black ink. The second page has X’s printed red, blue or green and the third page has colour names printed in contrasting colours; for example, the word “green” printed with red ink. The assessor will ask the respondent to read the words on page one or name the colours on pages 2 and 3 as quickly as possible within a 45 s time limit. The instructions for pages 2 and 3 require the respondent to inhibit the automatic response to say the word and instead name the colour. Scores are compared to normative data

### Health-related quality of life questionnaires

1. *Joint pain and function* will be assessed using the Western Ontario and McMasters University Osteoarthritis Index (WOMAC) [34]. The WOMAC is a self-assessed disease-specific measure of patients with OA of the hip and knee. This index assesses the severity of the knee pain during five daily activities and the severity of impairment of lower-extremity function during 17 activities. The items are scored with the use of a 10-cm Visual Analogue Scale, where 0 represents no pain or difficulty with physical function, with higher scores representing worse functional health
2. *Quality of life* will be measured using the self-administered Assessment of Quality of Life (AQoL) utility instrument, which assesses quality of life over five domains including illness, independent living, social relationships, physical senses and psychological wellbeing [35]. The AQoL instrument assesses the quality of life over five domains including illness, independent living, social relationship, physical senses and psychological wellbeing. The utility score for each dimension and an overall utility score range from 0 and 1, with 0 representing the worst health and 1 representing perfect health
3. *Physical activity level* will be assessed using The Incidental and Planned Exercise Questionnaire (IPEQ) for older people [36]. The IPEQ includes 10 questions that estimate physical activity during the last week which cover the frequency and duration of planned activity (planned exercise and walks) and incidental activities (casual day-to-day activities). The score was derived from multiplying the frequency score and duration score to create a total duration for incidental and planned activity as well as an overall total score. Total time spent will be summed across all components and expressed as hours per week as detailed in Delbaere et al. [36]
4. *Fear of falls* will be assessed using the Falls Efficacy Scale-International (Short FES-I) questionnaire [37]. The FES-I consists of seven items on Likert scale that score the participant’s level of concern regarding the possibility of falling when performing certain daily activities. The total score ranges from 7 (not concerned) to 28 (severe concern)
5. *Participant satisfaction* with the exercise program will be assessed at the completion of the 8-week exercise program using the Short Assessment for Patient Satisfaction measure (SAPS) [38]. The SAPS is a seven-item instrument that is used to assess patient satisfaction with their treatment. For the purposes of the present study, the phrases “treatment/care” will be changed to “exercise program” and “physician/other health professional” will be changed to “exercise leader/instructor.” The instrument has a 5-point Likert scale format, ranging from “very satisfied” to “very dissatisfied” or “strongly disagree” to “strongly agree.” Overall score ranges between 0 and 28 when 0 represents dissatisfaction and 28 represents great satisfaction

## Statistical analysis approach

Data collected for feasibility and safety will be analyzed using descriptive statistics (mean, standard deviation, and proportion) as follows: the percentage of participants who complete the intervention, overall percentage of sessions attended, number of participants who dropout, number of sessions and or number of exercises stopped due to pain, and any adverse event or incident that prevented the participant from completing the prescribed exercise.

Reported pain level will also be analyzed using descriptive statistics as follows: pain level during the exercise program, 2 h and 24 h after a session, any substantial increase in pain level to more than 7/10 or increase by 3 points, and overall pain level before, and at the completion of, the program.

To determine statistical trends of effectiveness, analyses of the selected outcomes, such as pain, strength, balance, physical function and executive functions, will be performed to assess the changes within and between groups over time (pre-post). Therefore, repeated measure analysis of variance (ANOVA) with factors of intervention (HSR, HSRTB, control) and time (pre-post intervention) will be used. This data will be used to determine power calculations for a fully powered future trial on the main outcomes investigated.

## Discussion

With the expected increase in the aging population and the number of people who suffer from knee OA, it is inevitable that falls and injuries experienced in this group will substantially rise. The reduction of mobility, injury and health-related quality of life associated with falls will have profound social and economic consequences into the future. The area of research in people with knee OA has been poorly studied despite the higher falls prevalence in this patient group [1–3]. The research project outlined in this protocol paper is the first step in addressing this deficit and will provide high-quality evidence for the safety and potential effectiveness of appropriate exercise programing for people with knee OA.

Balance exercises are key exercises recommended for fall prevention. Pain is a commonly reported barrier for not participating in exercise programs for people with OA; hence, carefully designing and evaluating an exercise intervention to address knee pain is crucial in order to develop safe, acceptable and effective fall-prevention interventions in this high-risk group. This study will be the first to provide outcomes that are critical to inform the development of an exercise-based fall-prevention intervention that is specifically designed to address the neuromuscular deficit associated with the increased risk factors for falls for people with knee OA. Consequently, the results of this pilot randomized controlled trial are essential to the design of a future, large, fall-prevention randomized controlled trial to reduce falls in this high-risk group.

### Trial status

Recruitment is ongoing.

## Additional file

Additional file 1: SPIRIT 2013 Checklist: recommended items to address in a clinical trial protocol and related documents. (DOC 123 kb)

## Abbreviations

1RM: One repetition maximum
AQoL: Assessment of Quality of Life
BW: Body weight
ESSA: Exercise and Sport Science Australia
FSST: The Four Square Step Test
HSRT: High-speed resistance training
HSRTB: High-speed resistance and balance training
IPEQ: The Incidental and Planned Exercise Questionnaire
NRS: Numeric Rating Scale
OA: Osteoarthritis
RPE: Rating of Perceived Exertion
SAPS: Short Assessment for Patient Satisfaction measure
Short FES-I: The Falls Efficacy Scale-International (short form)
SLUMS: Saint Louis University Mental Status
TMT: The Trail Making Test
WAIS-III: The Wechsler Adult Intelligence Scale-III
WOMAC: Western Ontario and McMasters University Osteoarthritis Index
